# Wiring Economy of Pyramidal Cells in the Juvenile Rat Somatosensory Cortex

**DOI:** 10.1371/journal.pone.0165915

**Published:** 2016-11-10

**Authors:** Laura Anton-Sanchez, Concha Bielza, Pedro Larrañaga, Javier DeFelipe

**Affiliations:** 1 Departamento de Inteligencia Artificial, Escuela Técnica Superior de Ingenieros Informáticos, Universidad Politécnica de Madrid, Madrid, Spain; 2 Laboratorio Cajal de Circuitos Corticales, Centro de Tecnología Biomédica, Universidad Politécnica de Madrid, Madrid, Spain; 3 Instituto Cajal, Consejo Superior de Investigaciones Científicas, Madrid, Spain; University Paris 6, FRANCE

## Abstract

Ever since Cajal hypothesized that the structure of neurons is designed in such a way as to save space, time and matter, numerous researchers have analyzed wiring properties at different scales of brain organization. Here we test the hypothesis that individual pyramidal cells, the most abundant type of neuron in the cerebral cortex, optimize brain connectivity in terms of wiring length. In this study, we analyze the neuronal wiring of complete basal arborizations of pyramidal neurons in layer II, III, IV, Va, Vb and VI of the hindlimb somatosensory cortical region of postnatal day 14 rats. For each cell, we search for the optimal basal arborization and compare its length with the length of the real dendritic structure. Here the optimal arborization is defined as the arborization that has the shortest total wiring length provided that all neuron bifurcations are respected and the extent of the dendritic arborizations remain unchanged. We use graph theory and evolutionary computation techniques to search for the minimal wiring arborizations. Despite morphological differences between pyramidal neurons located in different cortical layers, we found that the neuronal wiring is near-optimal in all cases (the biggest difference between the shortest synthetic wiring found for a dendritic arborization and the length of its real wiring was less than 5%). We found, however, that the real neuronal wiring was significantly closer to the best solution found in layers II, III and IV. Our studies show that the wiring economy of cortical neurons is related not to the type of neurons or their morphological complexities but to general wiring economy principles.

## Introduction

More than a century ago, Santiago Ramón y Cajal proposed the wiring economy principle. This principle states that neurons are arranged in such a way as to minimize the wiring cost where the structure of axons and dendrites is designed to save space, time and matter [1]. The significance of the neuronal wiring cost hypothesis, regarded as underlying principles of brain morphology and organization, has been widely studied (reviewed in [2]). Some researchers have suggested that the organization of certain regions of the brain is related to the need to reduce wiring costs [3–5]. Other studies have constructed synthetic neuronal structures to show that optimal wiring explains dendritic branching patterns [6–8].

In a previous paper, we used a new approach based on graph theory and evolutionary computation techniques to study the wiring features of different types of cortical GABAergic interneurons [9]. We found that wiring was near optimal in most of the tested dendritic and axonal trees of the different types of interneurons that we examined, including Martinotti, large basket, common type, horse tail, chandelier and common basket cells. These GABAergic interneurons account for no more than a minority of all neurons in the cerebral cortex and are genetically, molecularly, anatomical and physiologically distinct from pyramidal cells, the most abundant type of neuron in the cerebral cortex [10–14].

As a general rule, total wiring length refers to the sum of lengths of all axonal connections in a neural network. In this study, however, unless otherwise specified, wiring length refers to the branching structure of the dendritic arborization (branching points and terminal points of the dendritic trees). This is based primarily on previous studies analyzing whether the dendritic trees connect synaptic inputs to the dendritic root using the minimal total wiring length (see [6] as an example in the fly visual system). Here we analyze the neuronal wiring of individual pyramidal cells to check if this type of neuron also optimizes brain connectivity in terms of neuronal wiring cost. We express this cost as a function of the distance between elements (branching points and terminal points of the dendritic trees), because the cost of the connection increases as the distance between two elements widens. Since it is well established that pyramidal cell structure varies between different cortical areas and species (see [15] and [16] for reviews), our study focuses on the hindlimb somatosensory cortex of Wistar rats at postnatal day 14. Furthermore, we used this experimental animal and at this age since we intended to integrate these data with other anatomical, molecular, and physiological data that have already been collected from the same cortical region of the postnatal day 14 Wistar rats. The final goal is to create a detailed, biologically accurate model of circuitry across all layers of the primary somatosensory cortex within the framework of the Blue Brain Project [17].

Unfortunately, current methodological limitations restrict the analysis to either the complete basal arbors (horizontal sections) or truncated apical and basal arbors (coronal sections) of pyramidal cells. For the sake of consistency with our previous studies, we opted to study the basal dendrites first. Therefore, we investigated the dendritic architecture of complete basal arbors of pyramidal neurons in all cortical layers (layers II, III, IV, Va, Vb and VI) as it has been shown that dendritic morphologies are statistically different in each cortical layer [18]. Thus, we were also interested in examining whether, within a cortical area, there are possible differences in wiring optimality across all cortical layers.

## Materials and Methods

### Tissue preparation and cell reconstruction

**Fig 1:**
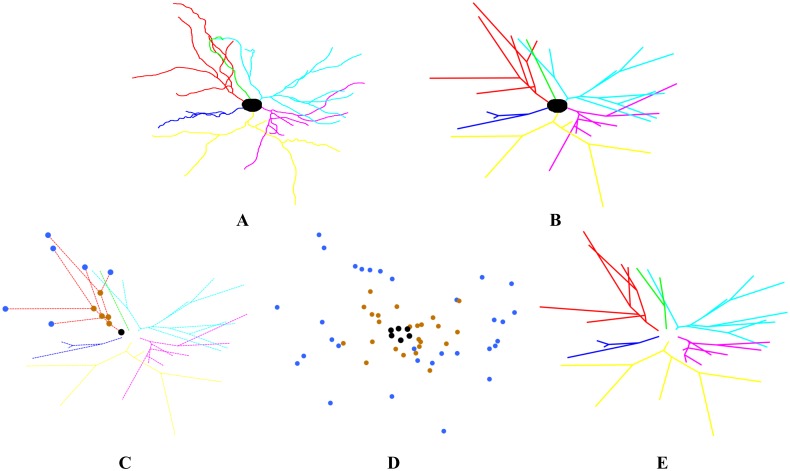
Example of one basal dendritic arbor of a pyramidal cell in layer II. A: Real 3D Neurolucida reconstruction where each dendritic tree is shown in a different color. B: Simplified real dendritic arbor where all connections are drawn as straight lines as we measure the (straight) length between points. C: Example of the identification of the root (black), branching points (brown) and terminal points (blue) of one dendritic tree. D: Point cloud formed by all the roots, branching and terminal points of the six basal dendritic trees. E: Shortest arborization found for the point cloud shown in D.

We analyzed the neuronal wiring of 288 3D-reconstructed complete basal arborizations of pyramidal cells across cortical layers II, III, IV, Va, Vb and VI of the somatosensory neocortex of the P14 rats (48 cells per layer). These basal dendritic arbors are made up of several main trunks, which are in turn composed of several dendrites. For the sake of simplicity and unless otherwise stated, we refer to these single trunks of basal dendritic arbors as *dendritic trees* (Fig 1). Data on the reconstruction and dendritic structure of these cells has been already published [18].

Briefly, Wistar rats (*n* = 20) were intraperitoneally injected with an overdose of sodium pentobarbitone and intracardially perfused with 4% paraformaldehyde. Their brains were then removed and sectioned with a vibratome. Sections (150–200 *μ*m) were cut parallel to the cortical surface and related to coronal sections. Using cytoarchitectural differences, we were thus able to identify the section that contained each cortical layer (II, III, IV, Va, Vb, VI) and then inject cells in the hindlimb somatosensory cortical region (more or less equivalent to the S1HL area in [19]). To this end, cells were labeled with 4.6 diamino-2-phenylindole (DAPI; Sigma, St Louis, MO) and then individually continuous current injected with lucifer yellow (LY) until the distal tips of each dendrite fluoresced brightly, indicating that the dendrites were completely filled and ensuring that the fluorescence did not diminish at a distance from the soma. After neuron injection, the sections were processed with a rabbit antibody to LY and thereafter treated with a biotinylated donkey anti-rabbit secondary antibody (1:200; RPN1004; Amersham Pharmacia Biotech), followed by a biotin–horseradish peroxidase complex (1:200; RPN1051; Amersham). Finally, 3.3’-Diaminobenzidine (DAB; D8001; Sigma Chemical Co.) was used as the chromogen, allowing the visualization of the entire basal dendritic arbor of pyramidal neurons. The Neurolucida package (MicroBrightField) was used to three-dimensionally trace the basal dendritic arbor of each pyramidal cell. Only neurons that had an unambiguous apical dendrite and whose basal dendritic tree was completely filled and contained within the section were included in the analysis. Animal housing, handling, experimental procedures, anesthesia and euthanasia conformed to all the terms and codes of ethics included in Spanish and European legislation (European Convention of Council of Europe ETS 123, Spanish Royal Decree 53/2013 and Law 6/2013, European Directive 63/2010/EU). This study was approved by the Ethics Committee of the Animal Facility of the Instituto Cajal of Madrid (Spain; registration number ES280790000184).

### Wiring analysis

We analyzed the neuronal basal wiring of single pyramidal neurons following the procedure described in [9] for dendritic wiring.

For each neuron, we started from a point cloud formed by the roots, branching points and terminal points of the real neuron and searched for the optimal dendritic arborization. This is defined as the arborization that has the shortest total length, subject to the output structure retaining the roots and terminal points of real neuronal trees. In addition, the output structure also contained the same number of branches of each real branching point. Then we compared the output minimal wiring length with the real wiring length. We used an approximate wiring length in both the real and output tree structures, because we measured the Euclidean distance between two connected points, that is, we ignored the path tortuosity.

Fig 1 illustrates this procedure with a pyramidal cell from layer II (all the neurons analyzed in this study are shown in [18]). The basal dendritic arbor of the neuron in Fig 1 has six main dendritic trees (shown in different colors in Fig 1A). Fig 1B shows the simplified dendritic arbor where all connections are straight lines with a total wiring length of 1954.97 *μ*m. For each dendritic tree, we identify the root of the tree, the branching points and the terminal points. Fig 1C shows the three types of points in the red tree (the root is shown in black, the branching points in brown and the terminal points in blue). We form the point cloud (Fig 1D) with the roots, branching and terminal points of all dendritic trees. We search for the minimum length arborization going through the above points. Fig 1E shows the best (shortest) structure found for this neuron, with a total length of 1912.79 *μ*m (2.16% shorter than the real wiring in Fig 1B). Note that since the roots are unchanged, the number of constructed trees always matches the number of trees in the real neuron. The branching and terminal points of different dendritic trees can be combined to arrive at an arborization with minimum length wiring.

To get the shortest arborization, we formulated the search for the optimal wiring of each neuron as a combinatorial optimization problem. We used genetic algorithms [20] to tackle this problem. Genetic algorithms are a heuristic technique providing approximate solutions. Specifically, we used the steady-state genetic algorithm [21] through graph theory with a novel permutation-based representation reported in [22]. Due to the stochasticity of genetic algorithms, we repeated the search for the optimal dendritic wiring 20 times for each cell and then chose the best (shortest) structure found for comparison with the real dendritic arborization.

## Results

**Fig 2:**
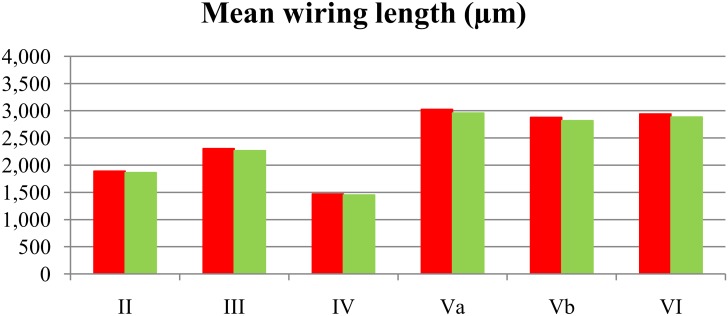
Mean wiring length (real vs. optimized). Mean wiring length (*μ*m) of the 48 analyzed cells in each cortical layer (red) versus mean wiring length of the shortest arborizations found by our optimization algorithm for each layer (green). The optimization algorithm found an equal or slightly better (shorter) wiring for all the neurons in all the layers. We found the biggest difference with respect to the real wiring in layer Va, where the synthetic wiring was, on average, 2.06% shorter than the real wiring. The smallest difference occured in layer IV, where the optimized wiring was, on average, 1.01% shorter than the real wiring.

The dendritic structure of the 288 analyzed cells are described in detail in [18]. The mean length of basal dendritic wiring, in microns, grouping the cells by layer is shown in red in Fig 2. This figure shows that, on average, the wiring of the neurons in layers Va, Vb and VI is longer than the neurons belonging to the three more superficial layers.

The mean number of trees in the basal arborizations of the 48 cells analyzed in each cortical layer ranges from 4.96 (layer IV) to 7.48 (layer VI), while the mean number of points in these arborizations is between 42.67 (layer IV) and 66.67 (layer Va) (Table 1). To check whether there were significant differences between layers, we performed a multiple mean comparison test on the number of trees and the number of points. First, we checked if the necessary assumptions to apply ANOVA were satisfied, i.e., if data were normally distributed (Kolmogorov-Smirnov test) and if homoscedasticity was met (Levene’s test). For the number of trees, none of the above assumptions were met, on which ground we used the Kruskal-Wallis test. The resulting p-value was 3.605e-12, i.e., there were differences in the number of trees between layers. Then, we applied the Mann-Whitney test with the Bonferroni method to adjust the p-values for pairwise comparisons. We found that there were differences between the number of trees in layers II vs. Vb, II vs. VI, III vs. IV, III vs. VI, IV vs. Va, IV vs. Vb and IV vs. VI. For the number of points in the arborizations of each layer, data were normally distributed but homocedasticity was not met. Therefore, we again applied the Kruskal-Wallis test and found significant differences between layers (p-value = 3.05e-11). In pairwise comparison, we found differences between layer IV and all the remaining layers.

**Table 1:** Mean and standard deviation (${\bar{x}}_{\pm s}$) of the number of dendritic trees and the number of points of the dendritic point clouds (roots, branching points and terminal points) of the 48 cells of each cortical layer.

Layer	Trees	Points
**II**	5.50_±1.01_	61.98_±9.19_
**III**	5.94_±1.29_	64.04_±10.56_
**IV**	4.96_±1.20_	42.67_±15.67_
**Va**	6.52_±2.04_	66.67_±22.12_
**Vb**	6.75_±1.72_	60.63_±17.70_
**VI**	7.48_±2.03_	62.75_±18.82_

We computed the optimal wiring length of the 288 cells. In order to compare the optimized wiring with the real wiring of each neuron, we calculated the percentage resulting from dividing the length of the shortest solution found by the real neuronal length. A figure of 100% shows that the length of the best solution found by our algorithm is equal to the total real wiring length of the neuron. A figure below 100% denotes that the solution found is better (shorter) than the real length, while a figure above 100% shows that the optimization algorithm is not able to improve the real dendritic tree.

**Fig 3:**
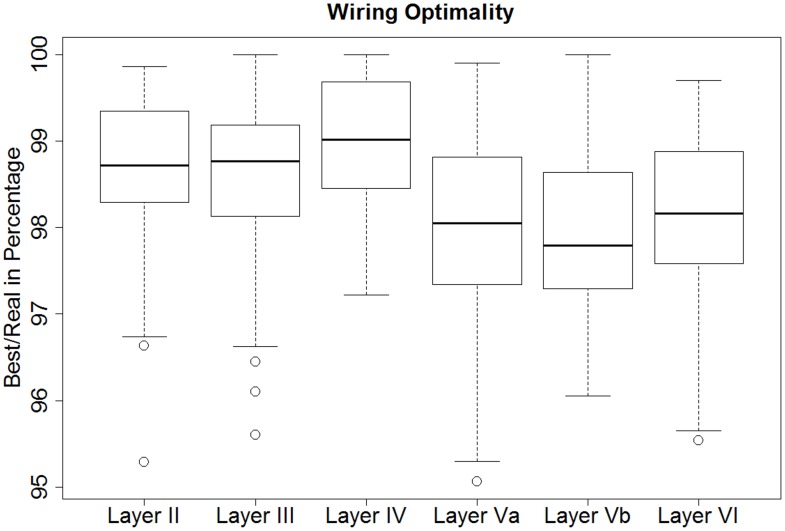
Box plot of the wiring analysis results for all layers. All the solutions are equal to or less than 100%, signifying that the solutions found by the optimization algorithm had a length equal to or shorter than the real wiring of the cells. For layers II, III and IV, the real neuronal wiring was closer to the shortest solutions found. Deeper layers had a higher degree of dispersion (steeper spacing between the parts of the box).

Fig 3 shows the box plot of the results. We found a shorter wiring than the real wiring for all neurons, except for one neuron in layer IV, for which the best solution found matched the real situation, that is, the percentage optimality for this neuron was 100%. A neuron in layer Va was found to have a wiring length that was nearly 5% shorter than the real one (95.06%, best result found). The percentage optimality for the remaining neurons ranged from 95.06% to 100%. The mean wiring length of the best solutions found for the 48 cells of each layer is shown in green in Fig 2.

**Fig 4:**
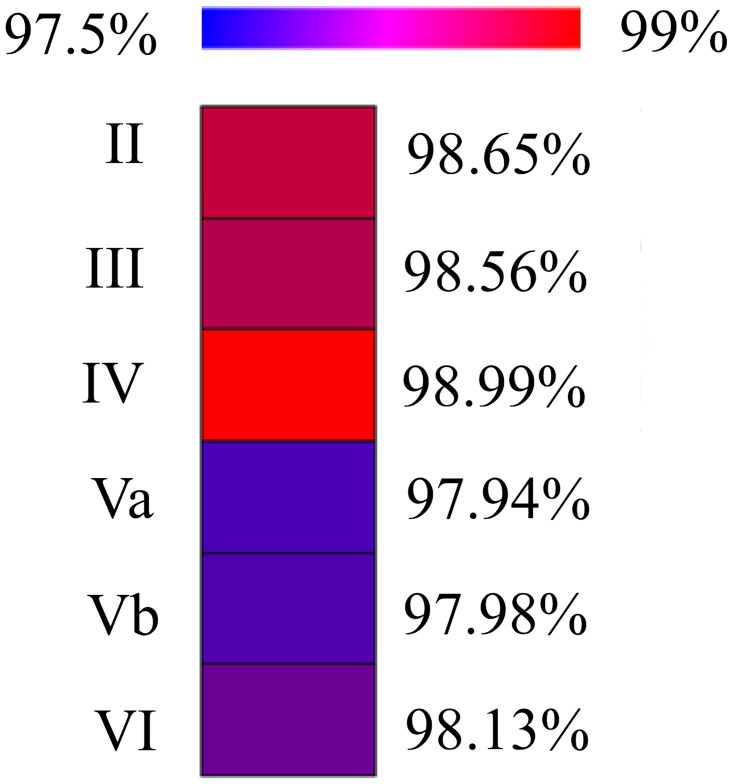
Mean optimality percentages of each cortical layer. Figures closer to 100% denote that the real neuronal wiring was closer to the shortest solutions found.

Fig 4 shows the mean percentage optimality for the neurons of each layer. Bluish colors denote that the wiring length of the best solutions found was further removed from the real wiring length, i.e., represented structures that offer a bigger improvement on the real neuronal structures. Reddish colors show that the total length of the resulting solutions was closer to the real neuronal length. Note, however, that the results ranged from 97.94% in layer Va to 98.99% in layer IV. Accordingly, all the results were very close to 100%.

We analyzed whether there were any significant differences in the wiring optimality, grouping neurons by layer. Since the data were normally distributed and homoscedasticity was met, we applied ANOVA. The resulting p-value was 1.54e-08, i.e., we rejected the null hypothesis of equal optimality in all six cortical layers. To find the differences between groups, we ran Tukey’s HSD (honest significant difference) test to evaluate all pairwise comparisons. The results showed that there were significant differences between the following pairs of layers: Va vs. II, Va vs. III, Va vs. IV, Vb vs. II, Vb vs. III, Vb vs. IV and VI vs. IV. Analyzing the results, we concluded that the behavior of our algorithm by layers could be divided into two groups: (i) layers II, III and IV (reddish in Fig 4) and (ii) layers Va, Vb and VI (bluish in Fig 4). We grouped cells accordingly and found that the percentage optimality of the second group (layers Va, Vb and VI) was significantly lower.

## Discussion

We analyzed the neuronal basal wiring of single pyramidal neurons across cortical layers following the procedure described in [9] for dendritic and axonal wiring optimization of GABAergic neurons. The interneurons discussed in [9] had rather complex morphologies and they showed many different anatomical characteristics, whereas pyramidal cells represent a much more homogeneous population of neurons. Thus, we tested the hypothesis of optimal neuronal wiring in single pyramidal cells with this method which represents a different approach from previous research on neuronal wiring. Specifically, the method imposes constraints that provide realistic synthetic arborizations, that is, forces the synthetic wiring of a specific cell to pass through the branching points to reach the terminal points of this neuron. It also limits the number of times that the points branch out. With this procedure, we proved that we can explain the wiring economy of single pyramidal cells considering only one specific criterion, i.e., wiring length.

The morphological characteristics of the same 288 pyramidal cells were analyzed in [18], concluding that there is a systematic layer-specific variation of the basal dendritic pattern in pyramidal cells. More specifically, the branching structure of pyramidal cells became progressively larger and more complex from superficial to deeper layers, save for layer IV, which contained the simplest cells. Although the morphological characteristics are statistically different in each cortical layer, our study has found that basal wiring arborizations were near optimal in terms of wiring length in all cases (the biggest difference between the shortest solution found for a neuron and the length of its real basal wiring was less than 5%). However, there appears to be a relationship between dendrite complexity and wiring economy since the solutions for the most superficial layers found by our algorithm were closer to the real wiring in our study, that is, the real cells manage to grow more optimally if they have a simpler branching structure. More specifically for cells in layer IV, the simplest according to [18], the real and optimal neuronal wirings were closer than in other layers. Nevertheless, it is noteworthy that neuronal connectivity depends not only on the dendritic wiring; but also on the density of dendritic spines, at least in the case of pyramidal cells. This is because most synapses on pyramidal cells are on their dendritic spines, and there are variations in the density of spines [10]. However, we do not know whether or not the density of spines and wiring optimization are related. Thus, further studies should be performed to determine whether neurons with high densities of dendritic spines have more or less optimal wiring attributes compared to neurons with low densities of dendritic spines.

Our previous study on the wiring of GABAergic interneurons [9] concluded that dendritic wiring was near optimal in the tested neurons in spite of the clear morphological differences between Martinotti, large basket, common type, horse tail, chandelier and common basket cells. On the whole, our studies show that the wiring economy of cortical neurons is not related to the type of neurons or their morphological complexities but to general principles of wiring economy. Nevertheless, this rule seems to apply to dendrites in particular since the wiring length of axonal trees of interneurons was, albeit near optimal, less so than for dendrites. In addition, although the differences in the wiring optimality between the basal dendritic arbors of pyramidal cells in different layers were small, they were statistically significant. As a result, the real wiring of the analyzed cells was nearer optimal in layers II, III and IV, whose branching structures are less complex according to [18], than in the deeper layers. Therefore, although wiring economy seems to be the general rule of optimization for cortical neurons irrespective of their anatomical and functional features, other factors may have an influence on the growth of the neuronal arborizations. More specifically, as previously discussed in [9], the trajectory of cellular processes could have “obstacles”, like blood vessels and cell somata, that the dendrite and axon trajectories have to circumvent. The more obstacles there are, the greater the wiring cost would be. Therefore, less optimal wiring might be expected in regions with a higher density of blood vessels and neurons.

Further studies in other cortical areas, layers and species are necessary to examine whether: (i) wiring economy is applicable to the dendritic and axonal arborizations of other types of neurons, including the apical dendrites and axons of pyramidal cells, and (ii) what is the biological significance, if any, of the small differences in the wiring of the basal dendritic arbors between pyramidal cells in different layers identified by this study or between the dendritic or axonal arborization of certain types of interneurons.
